# Serum metabolites associate with physical performance among middle-aged adults: Evidence from the Bogalusa Heart Study

**DOI:** 10.18632/aging.103362

**Published:** 2020-06-01

**Authors:** Jovia L. Nierenberg, Jiang He, Changwei Li, Xiaoying Gu, Mengyao Shi, Alexander C. Razavi, Xuenan Mi, Shengxu Li, Lydia A. Bazzano, Amanda H. Anderson, Hua He, Wei Chen, Jack M. Guralnik, Jason M. Kinchen, Tanika N. Kelly

**Affiliations:** 1 Department of Epidemiology, Tulane University School of Public Health and Tropical Medicine, New Orleans, LA 70112, USA; 2 Department of Medicine, Tulane University School of Public Health and Tropical Medicine, New Orleans, LA 70112, USA; 3 Department of Epidemiology and Biostatistics, University of Georgia College of Public Health, Athens, GA 30606, USA; 4 Institute of Clinical Medical Sciences, China-Japan Friendship Hospital, National Clinical Research Center of Respiratory Diseases, Beijing, China; 5 Children’s Minnesota Research Institute, Children’s Hospitals and Clinics of Minnesota, MN 55404, USA; 6 Division of Gerontology, Department of Epidemiology and Public Health, University of Maryland School of Medicine, Baltimore, MD 21201, USA; 7 Metabolon, Inc., Morrisville, NC 27560, USA

**Keywords:** physical performance, biomarkers, gait, successful aging, grip strength

## Abstract

Age-related declines in physical performance predict cognitive impairment, disability, chronic disease exacerbation, and mortality. We conducted a metabolome-wide association study of physical performance among Bogalusa Heart Study participants. Bonferroni corrected multivariate-adjusted linear regression was employed to examine cross-sectional associations between single metabolites and baseline gait speed (N=1,227) and grip strength (N=1,164). In a sub-sample of participants with repeated assessments of gait speed (N=282) and grip strength (N=201), significant metabolites from the cross-sectional analyses were tested for association with change in physical performance over 2.9 years of follow-up. Thirty-five and seven metabolites associated with baseline gait speed and grip strength respectively, including six metabolites that associated with both phenotypes. Three metabolites associated with preservation or improvement in gait speed over follow-up, including: sphingomyelin (40:2) (P=2.6×10^-4^) and behenoyl sphingomyelin (d18:1/22:0) and ergothioneine (both P<0.05). Seven metabolites associated with declines in gait speed, including: 1-carboxyethylphenylalanine (P=8.8×10^-5^), and N-acetylaspartate, N-formylmethionine, S-adenosylhomocysteine, N-acetylneuraminate, N2,N2-dimethylguanosine, and gamma-glutamylphenylalanine (all P<0.05). Two metabolite modules reflecting sphingolipid and bile acid metabolism associated with physical performance (minimum P=7.6×10^-4^). These results add to the accumulating evidence suggesting an important role of the human metabolome in physical performance and specifically implicate lipid, nucleotide, and amino acid metabolism in early physical performance decline.

## INTRODUCTION

Age related declines in physical performance are common among older adults [1] and robustly predict frailty, sarcopenia, disability, fracture, falls, cognitive impairment, reduced quality of life, comorbid chronic health conditions, and all-cause mortality [1–3]. Gait speed and hand grip strength are simple and non-invasive measures of physical performance in aging adults. Although reduced gait speed and grip strength have been established as risk factors for adverse health outcomes, their underlying biological pathways remain largely unknown. Examination of the human metabolome, which reflects endogenous and exogenous processes and their interactions [4], provides a unique opportunity to identify small molecule biomarkers of physical performance. Identified metabolites may serve as clinically relevant biomarkers and prognostic indicators of future physical performance decline.

Metabolomics has previously been used to examine aging and frailty. A previous study that compared centenarians to elderly individuals identified phospho/sphingolipids as markers of healthy aging [5]. A longitudinal analysis conducted among Framingham Heart Study participants found that some longevity related metabolomic pathways associate with risk of common causes of death [6]. Findings from patients with breast cancer included frailty-associated changes in amino acid and phospholipid metabolism [7]. Additionally, research in elderly participants identified 15 markers associated with frailty and suggested that oxidative stress could be implicated in the development of frailty [8].

Physical performance metabolomics studies have also been conducted. Previous research has identified metabolites associated with gait speed [9–12], other gait parameters [10], grip strength [11], combined muscle mass and strength outcomes [13, 14], and Short Physical Performance Battery (SPPB) score [11]. Although these findings are promising, limitations of past work include the sole use of cross sectional examination without longitudinal follow up [10, 11, 13–16], sole use of targeted metabolomics approaches which are restricted to pathways of presumed biological relevance [10–12, 14–16], small numbers of metabolites tested [10–16], small sample sizes [13–16], and lack of adjustment for multiple comparisons [10, 11, 14, 16]. Thus, there is a compelling need for research on the relationship between serum metabolites and physical performance that takes advantage of longitudinal data and utilizes agnostic metabolomic methods.

Our study was designed to fill this gap in knowledge by first examining the cross sectional relationships between serum metabolites identified through untargeted metabolomics and physical performance measures while utilizing stringent control for multiple testing. We next examined the relationships between identified metabolites and prospective declines in physical performance observed at a subsequent study visit. The Bogalusa Heart Study (BHS) was selected as the study population to increase generalizability and reproducibility of study findings through the use of a large, ethnically diverse, community-based sample and to identify metabolites that are relevant in middle age, prior to onset of age related sarcopenia or mobility disability.

## RESULTS

### Participant characteristics

The BHS is a community-based long-term study investigating the natural history of CVD among a multi-ancestry sample (35% black and 65% white) of residents from Bogalusa, Louisiana [17]. The current BHS population includes 1,298 participants born between 1959 and 1979 who were screened at least two times during childhood and two times during adulthood. Participant characteristics are presented in Table 1. Participants were mostly middle aged adults (mean age: 48.2) and obese [mean body mass index (BMI)>30]. Over 60% were hypertensive, while fewer than 20% had diabetes. Approximately 3% had chronic kidney disease. As was expected for the age and health status of the study participants, the cohort overall had high physical function at baseline, with a mean SPPB score of over 11 out of 12. Baseline gait speed and grip strength were modestly correlated (ρ=0.3, P<0.0001). The distributions of baseline gait speed and grip strength presented in Supplementary Figures 1, 2. Participants of the longitudinal study were similar to the entire cohort with respect to important prognostic indicators (Supplementary Table 1).

**Table 1:** Characteristics of BHS participants.

	**Overall (N=1,239)**	**Female**		**Male**	
		**Black (n=267)**	**White (n=463)**	**Black (n=160)**	**White (n=349)**
Age, years, mean (SD)	48.2 (5.3)	47.6 (5.4)	48.2 (5.1)	47.2 (6.0)	49 (5.0)
Post-high school education, n (%)	609 (49.2)	106 (39.7)	278 (60.0)	45 (28.1)	180 (51.6)
Smoking, n (%)					
Never	633 (51.1)	156 (58.4)	249 (53.8)	55 (34.4)	173 (49.6)
Former	362 (29.2)	67 (25.1)	138 (29.8)	48 (30.0)	109 (31.2)
Current	244 (19.7)	44 (16.5)	76 (16.4)	57 (35.6)	67 (19.2)
Drinking, n (%)					
Never	153 (12.4)	59 (22.1)	60 (13.0)	19 (11.9)	15 (4.3)
Former	395 (31.9)	81 (30.3)	149 (32.2)	49 (30.6)	116 (33.2)
Current	691 (55.8)	127 (47.6)	254 (54.9)	92 (57.5)	218 (62.5)
BMI, kg/m^2^, mean (SD)	31.5 (7.8)	34.9 (8.8)	30.2 (7.4)	31.2 (8.7)	30.5 (6.0)
SBP, mmHg, mean (SD)	123.3 (16.8)	125.6 (20.9)	117.3 (14.4)	131.2 (15.7)	125.8 (13.9)
Hypertension*, n (%)	771 (62.3)	196 (73.4)	225 (48.6)	127 (79.4)	223 (64.1)
Glucose, mg/dL, mean (SD)	107.6 (38.3)	108.3 (42.5)	105.2 (38.3)	110.5 (45.1)	109 (30.7)
Diabetes†, n (%)	207 (16.8)	47 (17.6)	73 (15.9)	29 (18.4)	58 (16.8)
eGFR, mL/min/1.73 m², mean (SD)	93.7 (17.0)	101.1 (18.4)	92 (14.2)	95.3 (20.3)	89.5 (15.7)
CKD (GFR‡<60 mL/min/1.73 m²), n (%)	39 (3.2)	7 (2.6)	14 (3.0)	6 (3.8)	12 (3.4)
SPPB score	11.1 (1.4)	10.7 (1.6)	11.2 (1.3)	10.8 (1.6)	11.2 (1.3)
Six-minute walk distance (m)	424.7 (86.1)	383.7 (72.6)	431.4 (81.7)	411.4 (81.5)	451.8 (90.8)
Gait speed (m/s)	1.2 (0.2)	1.1 (0.2)	1.2 (0.2)	1.1 (0.2)	1.3 (0.3)
Grip strength (kg)	35.1 (11.9)	28.6 (7.1)	27.2 (5.8)	44.9 (9.7)	46.1 (9.6)

Note. BHS=Bogalusa Heart Study, BMI=body mass index, CKD=chronic kidney disease, DBP=diastolic blood pressure, eGFR=glomerular filtration rate, SBP=systolic blood pressure, SD=standard deviation, SPPB=short physical performance battery.

· Hypertension was defined as SBP≥130 mmHg, DBP≥80 mmHg, or use of antihypertensive medication.

† Diabetes was defined as fasting plasma glucose≥126 mg/dL or use of diabetes medication.

### Metabolomics

Untargeted metabolomics resulted in the detection and relative quantification of 1,466 metabolites. These included 1,073 known biochemical compounds (Metabolomics Standards Initiative [MSI] levels 1 or 2) in pathways related to amino acids (n=201), carbohydrates (n=25), cofactors and vitamins (n=35), energy (n=9), lipids (n=435), nucleotides (n=42), peptides (n=52), and xenobiotics (n=256). An additional 18 partially characterized molecules (MSI level 3) and 393 unnamed compounds (MSI level 4) were also detected. The unnamed compounds may be identified upon the eventual acquisition of a matching purified standard (or via classical structural analysis). Of the metabolites examined, 1,202 metabolites passed rigorous quality control standards.

### Baseline overall analyses

Multivariable linear regression models were used to analyze associations between each metabolite and each baseline physical performance measure, after adjustment for age, gender, race, BMI, estimated glomerular filtration rate (eGFR), education, cigarette smoking, and alcohol drinking. Results of baseline multivariate adjusted linear regression models for gait speed and grip strength respectively are presented graphically as the magnitudes of P-values versus effect sizes (Supplementary Figure 3). Significant direct and inverse associations of metabolites with gait speed were identified, while significant metabolite associations with grip strength were all in the inverse direction.

### Baseline gait speed metabolite associations

**Figure 1:**
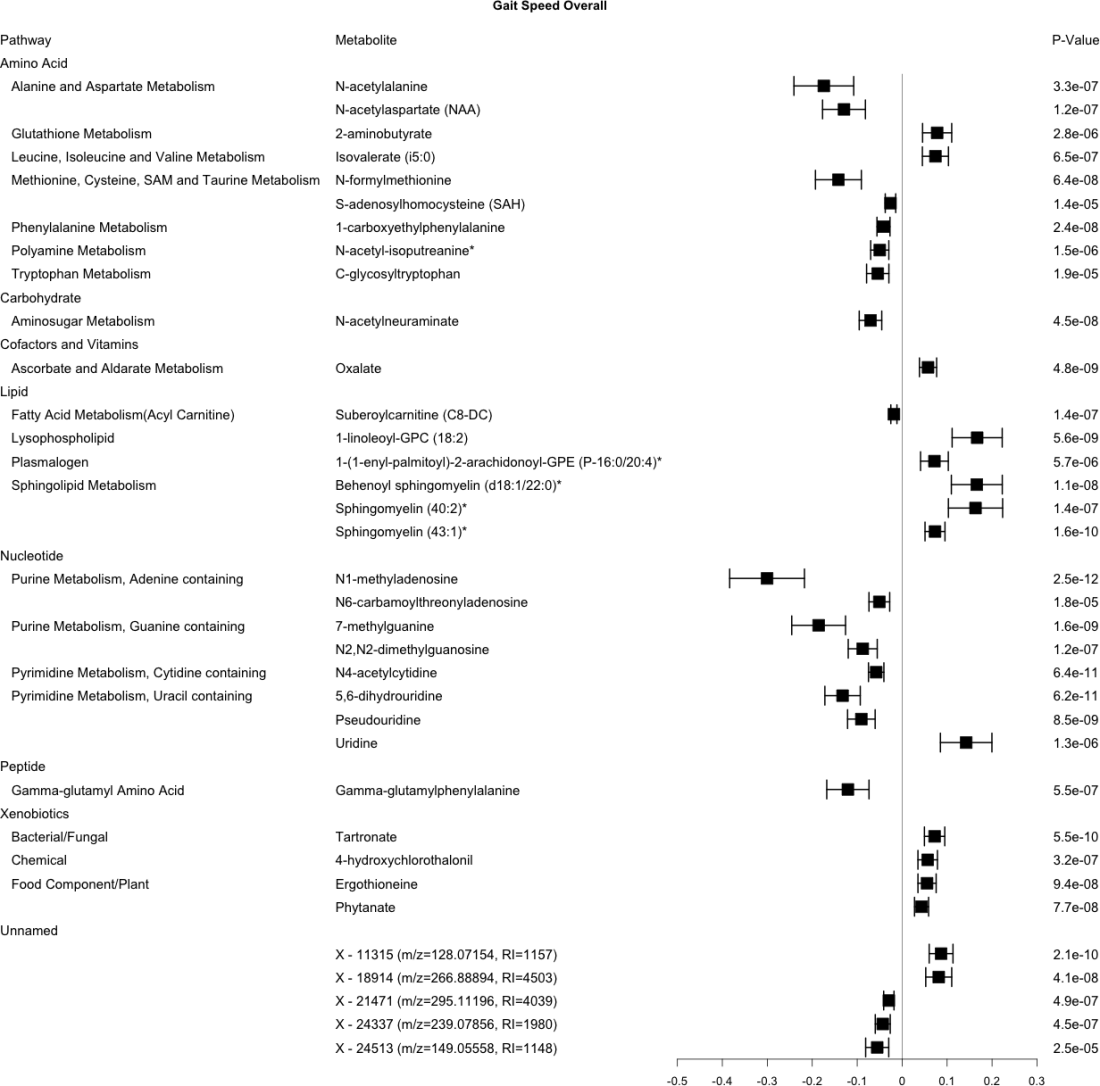
**Metabolites significantly associated with gait speed.** This forest plot depicts the beta estimate and 95% confidence interval for significant metabolites from both sex- and race-stratified analyses. 6 unknown metabolites are not shown. * indicates compounds with Metabolomics Standards Initiative confidence level 2.

In the cross-sectional analysis, 35 metabolites were robustly associated with gait speed, including 30 from the amino acid, carbohydrate, cofactor and vitamin, lipid, nucleotide, peptide, and xenobiotic pathways, and 5 unnamed compounds (Figure 1). Metabolites associated with gait speed, that internally replicated across sex or race are shown in Supplementary Figures 4–7. Results of a sensitivity analysis with additional adjustment for clinical covariates were generally consistent with our main findings for gait speed (Supplementary Table 2).

**Figure 2:**
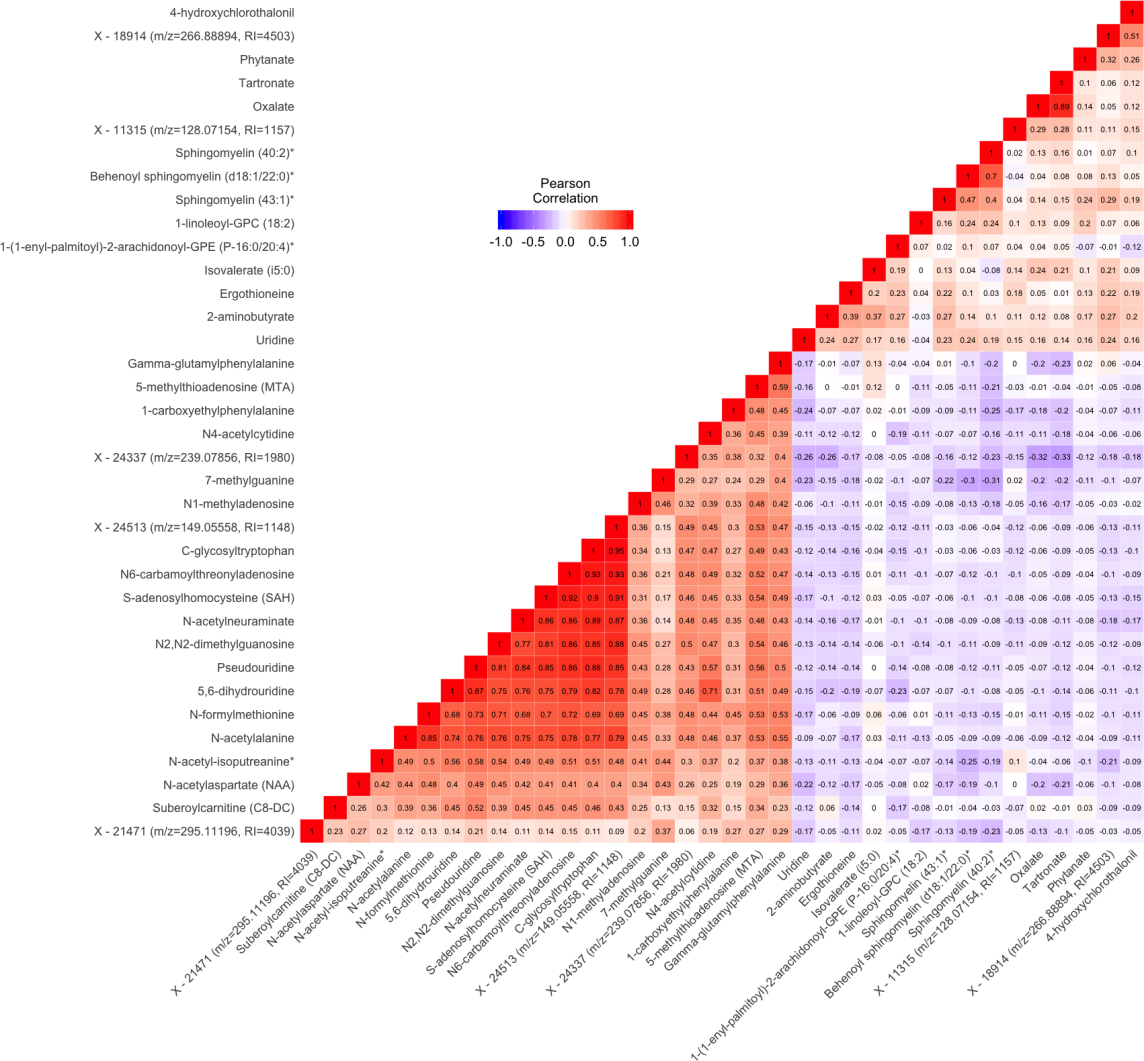
**Pairwise Pearson correlations between metabolites significantly associated with either gait speed or grip strength in cross sectional analysis.** Metabolites are ordered according to correlation coefficient. Correlations between each pair of metabolites are displayed in the cells of the heatmap. Cells are color coded with colors ranging from blue to red to depict correlations ranging from -1 to 1. RI=retention index. * indicates compounds with Metabolomics Standards Initiative confidence level 2.

There were notable correlations between groups of metabolites associated with gait speed (Figure 2). Most of the metabolites in the lipid pathway had positive associations with gait speed. The 3 metabolite signals in the sphingolipid pathway were modestly to highly

correlated (pairwise ρ ranging from 0.4 to 0.7). Additionally, oxalate from the cofactors and vitamins pathway and tartronate from the xenobiotics pathway were highly correlated (ρ=0.89). Most of the other pairs of metabolites positively associated with gait speed had lower correlations (ρ<0.3). Among the metabolites negatively associate with gait speed, a group of 10 were highly correlated [pairwise ρ ranging from 0.68 to 0.93, N-formylmethionine, N-acetylalanine, Pseudouridine, 5,6-dihydrouridine, S-adenosylhomocysteine, N6-carbamoylthreonyladenosine, C-glycosyltryptophan, N-acetylneuraminate, N2,N2-dimethylguanosine, and X - 24513 (m/z=149.05558, RI=1148)]. With some exceptions, lipid and xenobiotic metabolites tended to display modest negative correlations with the amino acid, carbohydrate, nucleotide, and peptide metabolites.

### Baseline grip strength metabolite associations

**Figure 3:**
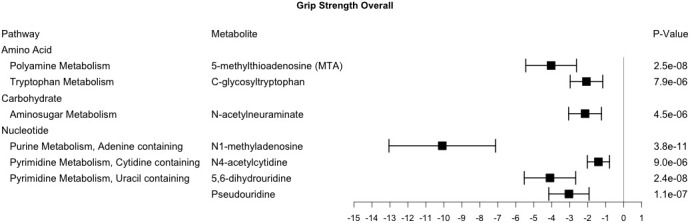
**Metabolites significantly associated with grip strength.** This forest plot depicts the beta estimate and 95% confidence interval for significant metabolites from both sex- and race-stratified analyses.

Cross-sectional study also identified 7 metabolites that were robustly associated with grip strength and included those from the amino acid, carbohydrate, and nucleotide (Figure 3). Six of these metabolites were also associated with baseline gait speed (C-glycosyltryptophan, N-acetylneuraminate, N1-methyladenosine, N4-acetylcytidine, 5,6-dihydrouridine, and Pseudouridine) and six had pairwise ρ>0.6 (C-glycosyltryptophan, N-acetylneuraminate, N1-methylinosine, N6-succinyladenosine, 5,6-dihydrouridine, and Pseudouridine) (Figure 2). Metabolites associated with grip strength, that internally replicated across sex or race are shown in Supplementary Figures 9–11. A sensitivity analysis with additional adjustment for clinical covariates generally produced results consistent with our main findings for grip strength (Supplementary Table 3).

### Longitudinal metabolite associations

After a mean (SD) follow up time of 2.9 (0.5) years, mean (SD) gait speed declined by 0.04 (0.20) meters per second. Mean (SD) grip strength declined by 0.6 (4.9) kg. Changes in gait speed and grip strength were not correlated (ρ=0.1, P=0.13). We found that 2 metabolites (out of the 35 from the baseline analysis) were associated with longitudinal change in gait speed at the Bonferroni corrected significance level (0.05/35=1.7×10^-3^). Despite the limited follow up time, 8 additional metabolites were nominally associated with change in gait speed (P<0.05) (Table 2), and 9 of the metabolites identified longitudinally had effect directions that were consistent with the baseline analysis (Supplementary Table 4). None of the 7 metabolites tested for association with longitudinal change in grip strength met nominal statistical significance thresholds. Even among null findings, effect directions for longitudinal change in gait speed and grip strength were generally consistent with effect directions identified in the cross-sectional analysis (Supplementary Table 5).

**Table 2:** Associations with longitudinal change in gait speed between baseline and follow up.

**Pathway**	**Metabolite**	**Beta (SE)**	**P-Value**
Positive in Cross-Sectional Analysis			
Lipid			
Sphingolipid Metabolism	Behenoyl sphingomyelin (d18:1/22:0)*	0.15 (0.06)	0.02
	Sphingomyelin (40:2)*	0.26 (0.07)	2.6×10^-4^
Xenobiotics			
Food Component/Plant	Ergothioneine	-0.05 (0.02)	9.6×10^-3^
Negative in Cross-Sectional Analysis		
Amino Acid			
Alanine and Aspartate Metabolism	N-acetylaspartate (NAA)	-0.13 (0.06)	0.04
Methionine, Cysteine, SAM and Taurine Metabolism	N-formylmethionine	-0.12 (0.05)	0.01
	S-adenosylhomocysteine (SAH)	-0.03 (0.01)	0.02
Phenylalanine Metabolism	1-carboxyethylphenylalanine	-0.09 (0.02)	8.8×10^-5^
Carbohydrate			
Aminosugar Metabolism	N-acetylneuraminate	-0.05 (0.03)	0.04
Nucleotide			
Purine Metabolism, Guanine containing	N2,N2-dimethylguanosine	-0.05 (0.03)	0.05
Peptide			
Gamma-glutamyl Amino Acid	Gamma-glutamylphenylalanine	-0.12 (0.05)	0.03

* Indicates compounds with Metabolomics Standards Initiative confidence level 2.

2 unnamed metabolites are not shown.

### Overlap with kidney function metabolites

The metabolites inversely associated with physical performance were all also associated with kidney function in our previous study in the BHS, with consistent effect direction [18]. These metabolites are presented in Supplementary Table 6, along with lookups of their associations with aging, inflammation, and mortality in previous research.

### Metabolite module associations

**Figure 4:**
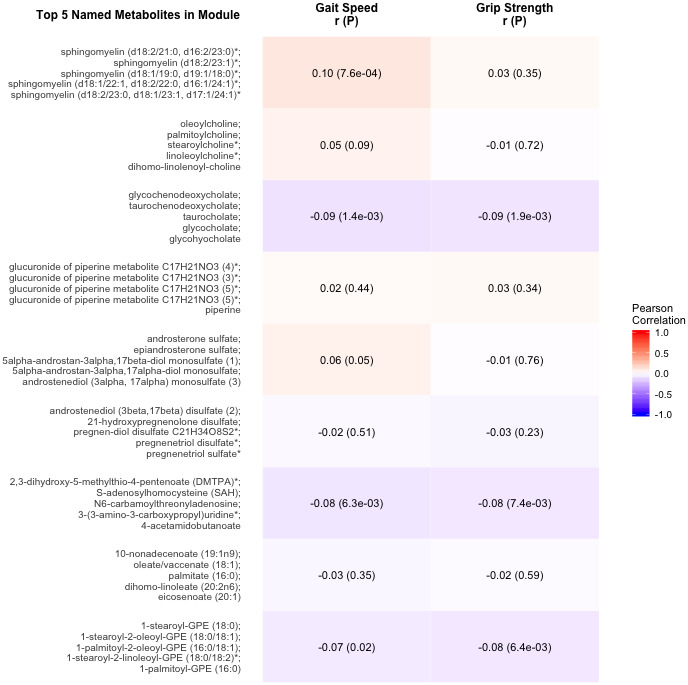
**Correlations of metabolite modules with physical performance.** Colors represent correlation strength, ranging from blue (-1) to red (1). * indicates compounds with Metabolomics Standards Initiative confidence level 2.

The 9 metabolite modules identified among BHS participants are shown in Figure 4. A module, with sphingolipids as top metabolites, was positively associated with gait speed (p=7.6×10^-4^). Another module, with top metabolites in the primary and secondary bile metabolism pathways (top metabolite: glycochenodeoxycholate), was negatively associated with both gait speed (p=1.4×10^-3^) and grip strength (p=1.9×10^-3^).

## DISCUSSION

This study examined cross-sectional and longitudinal associations of untargeted serum metabolites with two measures of physical performance, gait speed and grip strength. Thirty-five and 7 metabolites robustly associated with gait speed and grip strength, respectively. Six of these metabolites associated with both phenotypes, suggesting both common and disparate biological mechanisms underlying these physical performance phenotypes. Effect directions in longitudinal analyses were generally consistent with those of the cross-sectional study, with 2 metabolites achieving significant associations with change in gait speed after adjusting for baseline measures. No metabolites achieved significant longitudinal associations with grip strength. We additionally identified two metabolite modules associated with physical performance measures. Many of the identified metabolites represent novel findings in physical performance metabolomics literature, and implicate lipids, amino acids, and nucleotides in the physiologic processes related to this complex phenotype.

### Lipid metabolites

Sphingolipids, and a metabolite module with top metabolites in the sphingolipids pathway, were positively associated with gait speed in the current study. The three metabolite signals in this pathway identified in our study, behenoyl sphingomyelin (d18:1/22:0), sphingomyelin (40:2) and sphingomyelin (43:1), represent novel findings. Other sphingolipids, however, have previously been shown to associate with gait speed [10, 11] and other physical performance measures [11], adding credence to the robustness of our findings. Sphingolipids levels have also been shown to be decreased in multiple sclerosis [19], a disease characterized by demyelination, leading to axonal and neuronal loss [20] reductions in gait speed [21], and other gait abnormalities [21]. Previous research in older adults posits that sphingolipid metabolism may relate to physical performance similarly, through the insulation of nerve cell axons by myelin sheaths, thus influencing nerve conduction signals [11]. Additionally, sphingolipids have been previously associated with aging [22], neurodegeneration [23], and cognitive decline [24]. Further research is needed to elucidate whether decreased serum sphingolipid levels represent a potential novel clinical biomarker of demyelination in the general population that can be used to predict incident reductions in physical performance.

### Nucleotide metabolites

We identified several modified nucleosides with known relationships to whole body RNA degradation, including N2,N2-dimethylguanosine [25], 5,6-dihydrouridine [26], 7-methylguanine [25], N4-acetylcytidine [27], and pseudouridine [25]. The higher levels of these metabolites found in participants in our study with lower physical performance mirror the elevated levels of these metabolites in serum from human patients with pulmonary arterial hypertension [28] and end stage renal disease [27], urine from those with cancer [29], AIDS [29], and recent surgical stress [30], as well as urine from tree shrews with increased social stress [31]. Modified nucleosides enter circulation during stress [32], accelerated cell proliferation [29], and rapid tissue breakdown [29]. One potential hypothesis explaining the elevated levels of these metabolites in serum of participants with lower and declining physical performance is that the metabolites and lower physical performance measures are both markers of increased stress and tissue breakdown. While the modified nucleoside, pseudouridine, was inversely associated with gait speed (at baseline and longitudinally) and grip strength, its nucleoside precursor, uridine was directly associated baseline gait speed. Similarly to a metabolomics study of esophageal adenocarcinoma that found higher levels of pseudouridine and lower levels of uridine in cases compared to controls [33], one potential explanation is that participants with slower gait speed could have a higher rate of conversion of uridine to pseudouridine than those with faster gait speed. Further study is needed to examine whether these metabolites, along with reductions in physical performance, represent early biomarkers of future declines in health status.

### Kidney function and aging

Interestingly, all of the metabolites with inverse associations with physical performance measures were also inversely associated with kidney function in the BHS [18]. Many of these metabolites were also associated with kidney function in other populations [27, 34–37]. In prior study of a population with chronic kidney disease (CKD), CKD severity was associated with poor physical performance and frailty in a graded fashion [38]; however, the biological mechanisms leading to reduced physical function in CKD patients remain unknown. It is well known that small molecules accumulate in serum as kidney function declines [39]. One theory on the relation between kidney function and physical performance is that this accumulation of small molecules has a detrimental effect on other systems in the body [40], including those that govern physical performance – that declines in kidney function lead to declines in physical performance through serum metabolites. In support of this theory, two recent papers posit a relation between declines in kidney function and sarcopenia through serum metabolites [41, 42]; however, the extent to which these findings apply to other physical performance measures is unclear. An alternative theory is that declines in kidney function and declines in physical performance are both governed by a common ‘accelerated aging’ phenotype [38], and that identified common metabolites may be markers of accelerated aging. In our study, this theory is supported by the very low prevalence of CKD among the BHS population and the significance of the identified metabolites after adjustment for eGFR. This theory is additionally supported by shared risk factors between physical performance decline and CKD, such as lower socioeconomic status, lower physical activity, and increased rates of cardiovascular disease and cerebrovascular disease, chronic low-grade inflammation, and hyperglycemia [9]. In further support of this theory, several of our findings, that associated with both physical performance and kidney function, are also associated with aging [43–48], inflammation [49, 50], and mortality [51–55]. Additional research is warranted to examine the role of the identified metabolites in the relationship between reduced kidney function and declines in physical performance.

### Strengths and limitations

This study has several important strengths. The longitudinal design allowed us to identify metabolites that associated with future changes in physical performance, while adjusting for baseline performance variables. Metabolites identified longitudinally are more likely to be relevant to the development of physical performance declines than metabolites identified solely through cross sectional analysis. To our knowledge, this study had a larger baseline sample size than prior physical performance metabolomics research, increasing the power to detect metabolite-phenotype associations. Additionally, metabolites were detected using an untargeted approach, thus increasing the likelihood of finding novel signals. To reduce the likelihood of false positive associations and increase the generalizability of the findings, only signals that achieved significance in the overall sample and one gender or race group, with consistency in effect direction and nominal significance in the other gender or race group, were reported here. While this method should minimize spurious signals, it also prohibits the identification of gender- or race-specific findings. One notable limitation is that metabolites measured in middle-age could reflect the result of early life influences rather than directly influencing the aging process. Our study lacked external replication, however, we replicated our findings internally across gender or race. Other limitations include the relatively short mean follow up time of less than three years, use of a subset of the baseline sample for the longitudinal analysis, and the unknown clinical significance of the unnamed metabolites identified. Although these limitations reduced our power to detect longitudinal associations, several compelling temporal relationships were detected. Due to the age and overall high functioning status of the study participants, we were unable to examine physical performance using the SPPB, however, baseline SPPB information has been collected on these participants and changes in scores may be available for future studies in this population.

## CONCLUSIONS

In summary, we identified 36 metabolites cross-sectionally associated with physical performance measures, including 35 for gait speed and 7 for grip strength. Of these metabolites, 2 were longitudinally associated with declines in gait speed. These findings suggest important roles for sphingolipid metabolism and whole body RNA breakdown in declining physical performance in a middle-aged population.

## MATERIALS AND METHODS

### Participants

The BHS is a long-term study investigating cardiovascular health over the life-course. From 1973 to 2016, 7 surveys were conducted in children and adolescents aged 4 to 17 years, and 10 surveys were conducted among adults aged 18 to 51 years who had been examined previously as children. The BHS has been described in detail elsewhere [17]. Data and specimens collected in the recent 2013 to 2016 visit cycle were leveraged in our cross-sectional analysis and served as the baseline measures for longitudinal study of physical performance decline. Those missing baseline metabolomics (n=37), grip strength (n=17), gait speed (n=80) or covariable (n=17) data were excluded from all analyses. A total of 1,227 and 1,164 participants remained for the cross-sectional metabolomics study of grip strength and gait speed, respectively. The longitudinal study included 282 and 201 of these participants with repeated measures of grip strength and gait speed, respectively, which were collected an average of 2.9 years following the baseline examination.

Informed consent was obtained from all study participants after detailed explanation of the study. This study was conducted according to the principles expressed in the Declaration of Helsinki and was approved by the Tulane University Institutional Review Board.

### Metabolomics

Untargeted, ultrahigh performance liquid chromatography-tandem mass spectroscopy (UPLC-MS/MS) of BHS serum samples was conducted by Metabolon Inc. (Durham, NC) [56] using samples that were stored at -80°C since the 2013 to 2016 visit. Rigorous quality assurance was conducted, which included the use of blanks, blind duplicates (5% of samples), and standard biochemical compounds which were integrated into every run. Batch effects were assessed using principal components analysis, which revealed no evidence of clustering of metabolite data by run-days. A complete list of the metabolites examined and their properties is presented as Supplementary Data.

Similar to previous analyses [57], data filtering excluded 213 metabolites that were below the detection threshold in more than 80% of samples and 51 metabolites with a reliability coefficient <0.3 based on blind duplicate analysis. Among the 1,202 metabolites passing quality control, 167 metabolites were below the detection threshold in 50% to 80% of the samples, and were analyzed as ordinal variables after categorization into one of three mutually exclusive groups: 1) below-the-detection-limit; 2) below the median of measured values; or 3) greater than or equal to the median. The remaining 1,035 metabolites, which were above the detection threshold in more than 50% of samples, were analyzed as continuous variables, scaled to set the median of detected values for each metabolite equal to 1, where the minimum observed value was imputed for metabolites with below-the-detection-limit values.

### Outcomes and covariables

Among BHS participants, phenotype and covariable data were collected following stringent protocols [58]. Gait speed, in meters per second, was determined by dividing six-minute walking distance by 360 seconds. Grip strength was measured using a Jamar hand held dynamometer and was averaged across both hands. Questionnaires were administered to obtain information on demographic characteristics, lifestyle risk factors, and personal medical history. Anthropometric measures were obtained by trained staff with participants in light clothing without shoes. During each visit, body weight and height were measured twice to the nearest 0.1 kg and 0.1 cm, respectively. The mean values of height and weight were used to estimate BMI, which was calculated as weight in kilograms divided by height in meters squared. BHS participants were instructed to fast for 12 hours prior to blood sample collection. Serum creatinine level was measured by Laboratory Corporation of America (LabCorp, Burlington, NC) using the kinetic Jaffe method. Estimated glomerular filtration rate (eGFR) was calculated using the 2009 CKD-EPI equation [59].

### Statistical analysis

Characteristics of BHS participants were presented as means and standard deviations (SDs) for continuous variables and as percentages for categorical variables.

### Association of single metabolites with physical performance phenotypes

The two physical performance measures studied were gait speed and grip strength. Multivariable linear regression models (using SAS [version 9.4; SAS Institute, Cary, NC] function PROC GLM) were employed to analyze associations between each metabolite and each untransformed baseline physical performance measure, after adjustment for age, BMI, eGFR, education, cigarette smoking, and alcohol drinking. Analyses were performed according to gender and race, and in an overall analysis after additional adjustment for gender and race. All analyses accounted for multiple testing using the Bonferroni method. To further reduce false positive findings, we relied on internal replication across gender or race. Metabolites were considered robustly significant if they were significant in the overall analysis, and significant in either gender or race with a consistent effect direction and nominal significance (p<0.05) in the other gender or race. Pairwise Pearson correlations were calculated between significant metabolites for each physical performance measure, and heatmaps were created using the ggplot2 and reshape R (version 3.4.3) packages. To account for potential confounding by clinical factors, we conducted a sensitivity analysis with adjustment for fasting glucose, systolic blood pressure, and low-density lipoprotein, in addition to the covariates from the main analysis. We additionally examined the overlap in significant findings between this study and the findings of our previous kidney function metabolomics publication in the BHS [18].

Longitudinal changes in gait speed and grip strength were calculated by subtracting baseline measures from follow up measures. Multivariable linear regression models (using SAS function PROC GLM) were also employed to analyze the associations between each metabolite that was robustly significant in overall cross-sectional analysis and change in each physical performance measure, after adjustment for the appropriate baseline physical performance measure, follow up time, age, gender, race BMI, eGFR, education, cigarette smoking, and alcohol drinking. Due to the small sample size and criteria of consistency of associations across race or gender groups in cross-sectional analyses, longitudinal analyses were not stratified by gender or race.

### Association of metabolite modules with physical performance phenotypes

We used weighted correlation network analysis (WGCNA) [60] to identify networks of highly correlated metabolites. WGCNA is an unsupervised data reduction technique that allows for dependency between components, which may represent the biological pathways of identified metabolites more accurately than principal components analysis [60, 61]. The use of WGCNA and its application to metabolomics studies has been previously reported [62]. In brief, the metabolite network was constructed as an adjacency matrix based on the weighted pairwise correlations of all metabolites [63]. Modules, defined as densely interconnected metabolites, were then identified from the network using an unsupervised hierarchical clustering approach [64]. For each module, an eigenmetabolite was generated. This measure represents the module’s first principal component and can be interpreted as its weighted average metabolite value. Metabolite modules were constructed using metabolite data for the 1,202 metabolites passing quality control among all study participants.

Adjusted physical performance measures were created using the residual values generated by regressing each raw physical performance phenotype on gender, race, age, BMI, eGFR, education, cigarette smoking, and alcohol drinking. The correlations between each module (eigenmetabolite) and the adjusted physical performance phenotypes were then estimated. We employed a Bonferroni corrected alpha threshold of 5.56×10^-3^ (0.05/9) to account for testing 9 metabolite modules. WGCNA analysis was performed using the WGCNA R package. The figure depicting the WGCNA results was created using the ggplot2 R package.

## Supplementary Material

Supplementary Figures

Supplementary Tables

Supplementary Data
