# Supplementary material for: Serum metabolites associate with physical performance among middle-aged adults: Evidence from the Bogalusa Heart Study
Source: Aging (Albany NY). 2020 Jun 1;12(12):11914–41. doi: 10.18632/aging.103362 (PMC7343486; doi:10.18632/aging.103362)
Supplement: Supplementary Figures [file aging-12-103362-s003..pdf]

SUPPLEMENTARY FIGURES

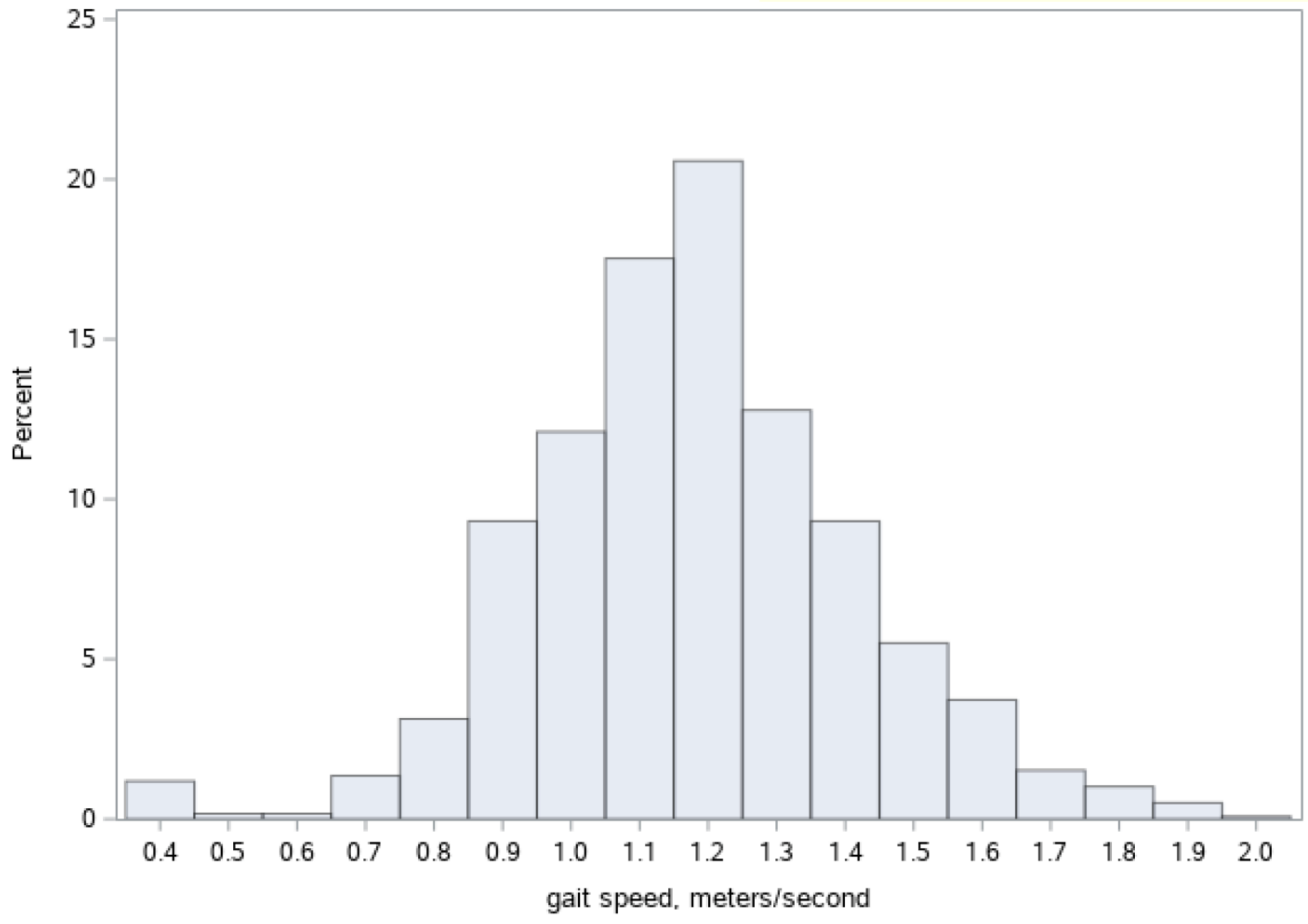

Supplementary Figure 1. Distribution of Baseline Gait Speed.

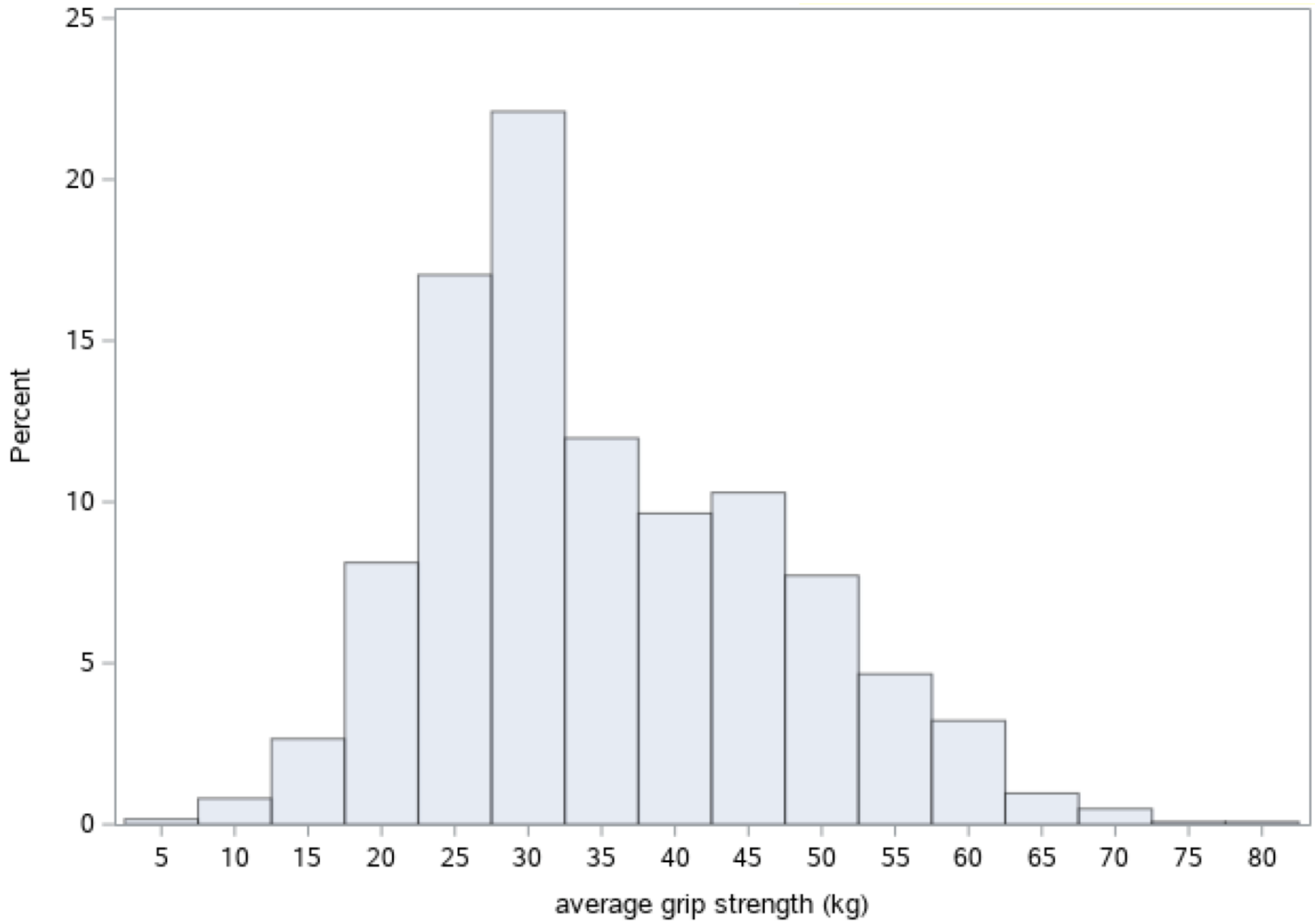

Supplementary Figure 2. Distribution of Baseline Grip Strength.

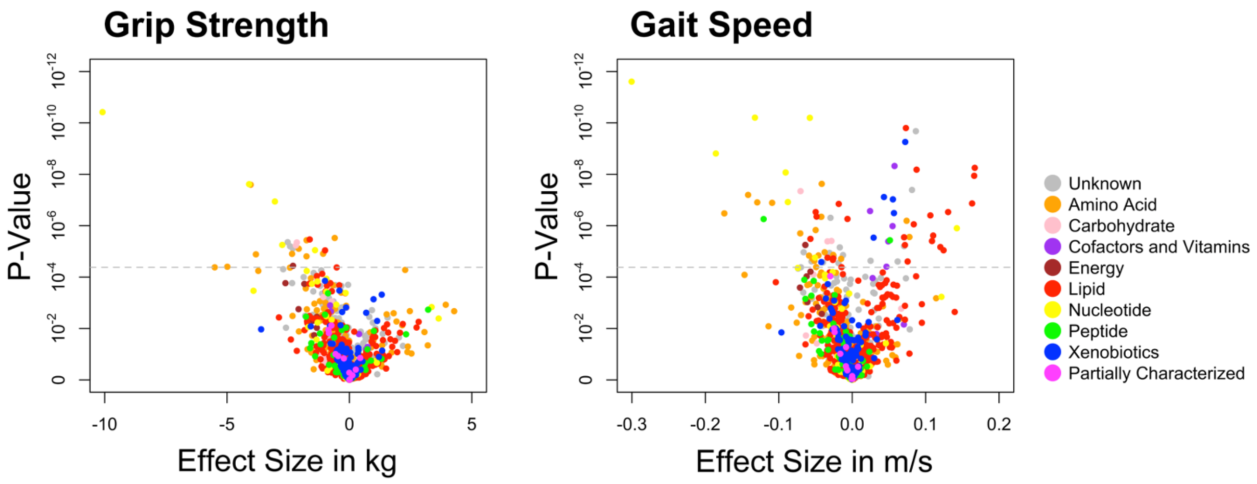

Grey=unknown; orange=amino acid; light pink=carbohydrate; purple=cofactor and vitamin; brown=energy; red=lipid; yellow=nucleotide; green=peptide; blue=xenobiotic; dark pink=partially characterized molecule.
BHS=Bogalusa Heart Study.
The grey dashed line indicates the Bonferroni corrected α threshold (0.05/1202=4.2×10-5) that was used for this study.

Supplementary Figure 3. P-values vs. effect sizes for grip strength and gait speed among BHS participants.

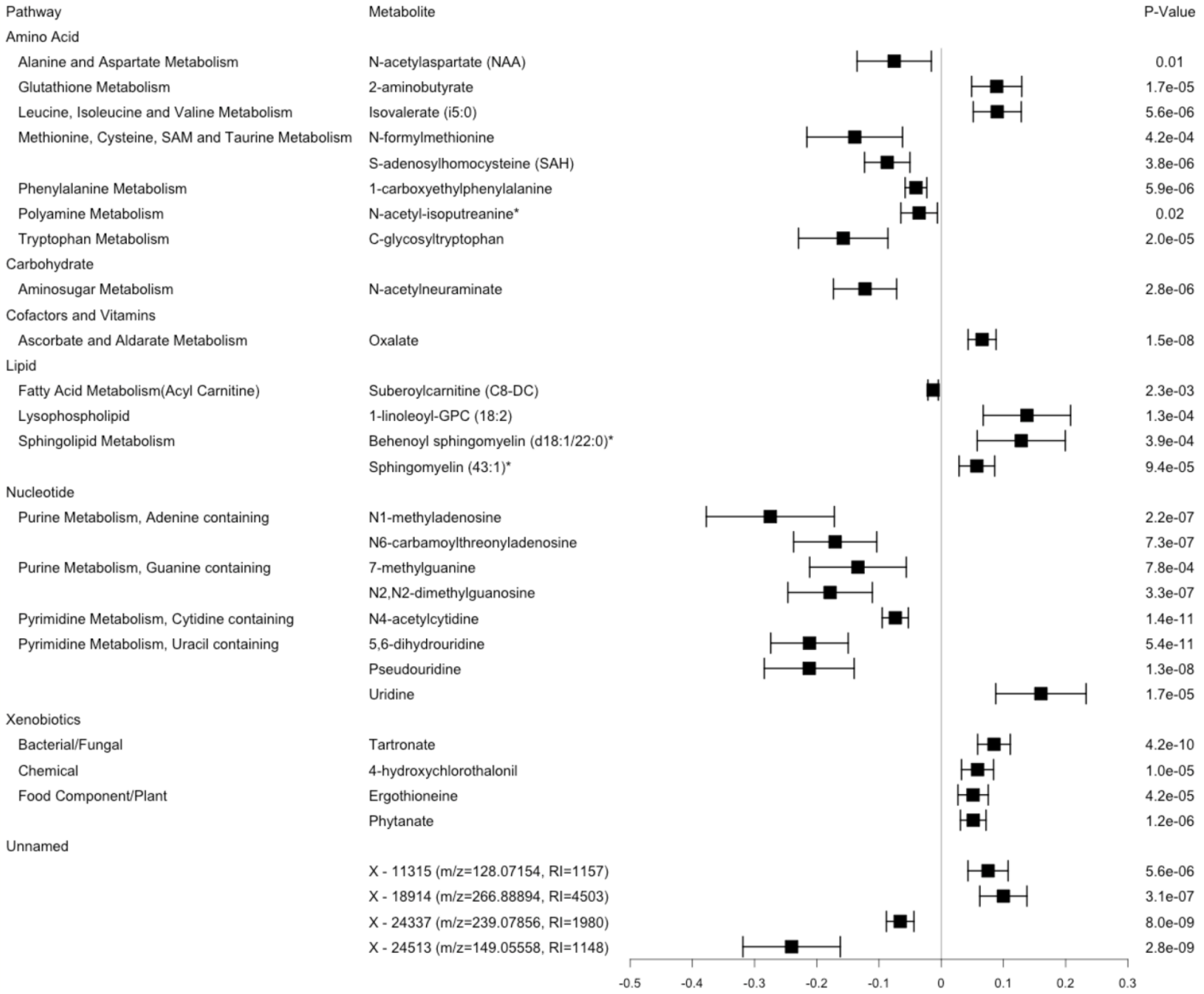

* Indicates compounds with Metabolomics Standards Initiative confidence level 2.

Supplementary Figure 4. Metabolites Significantly Associated with Gait Speed in Sex Stratified Analysis, among Female Participants.

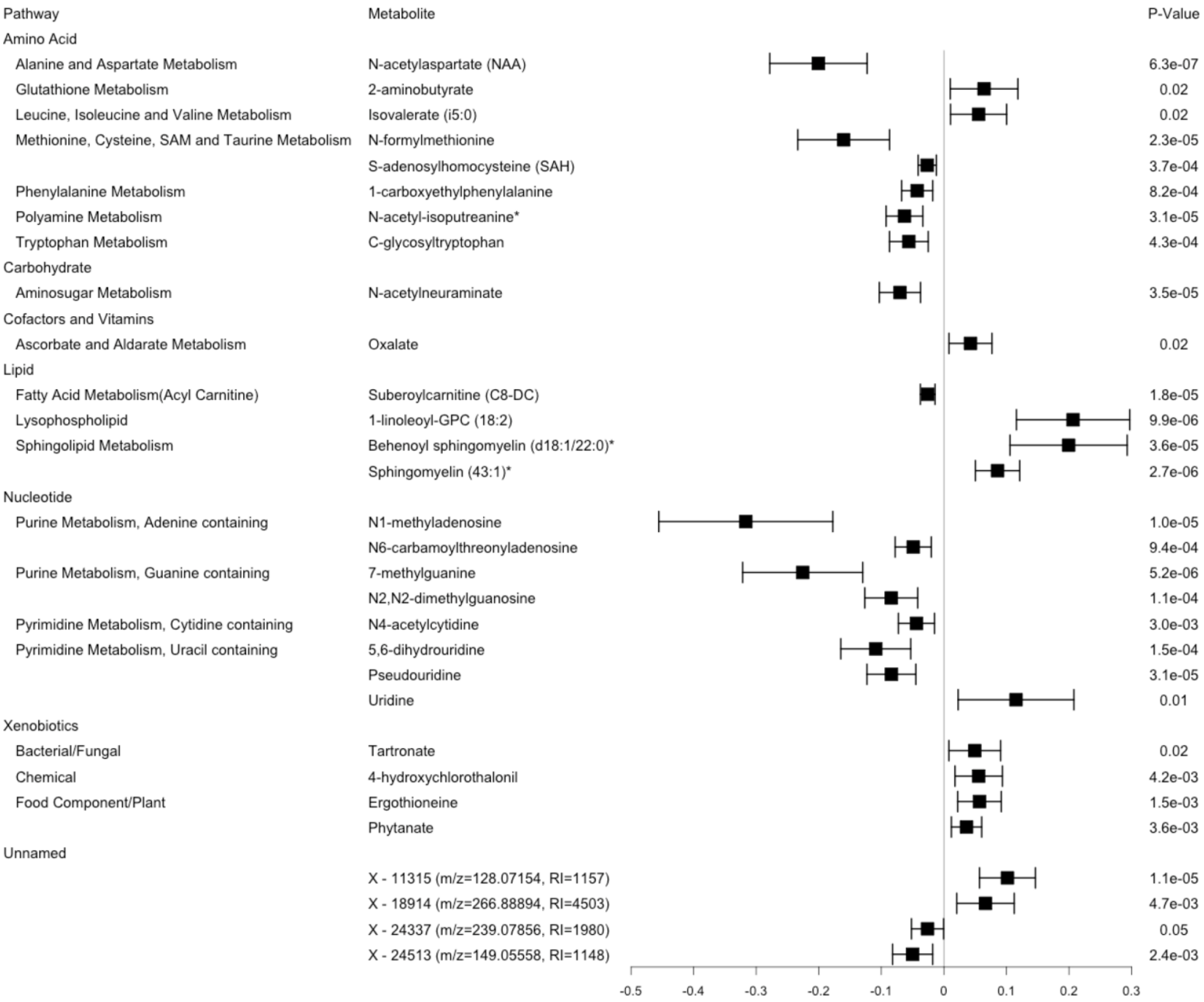

* Indicates compounds with Metabolomics Standards Initiative confidence level 2.

Supplementary Figure 5. Metabolites Significantly Associated with Gait Speed in Sex Stratified Analysis, among Male Participants.

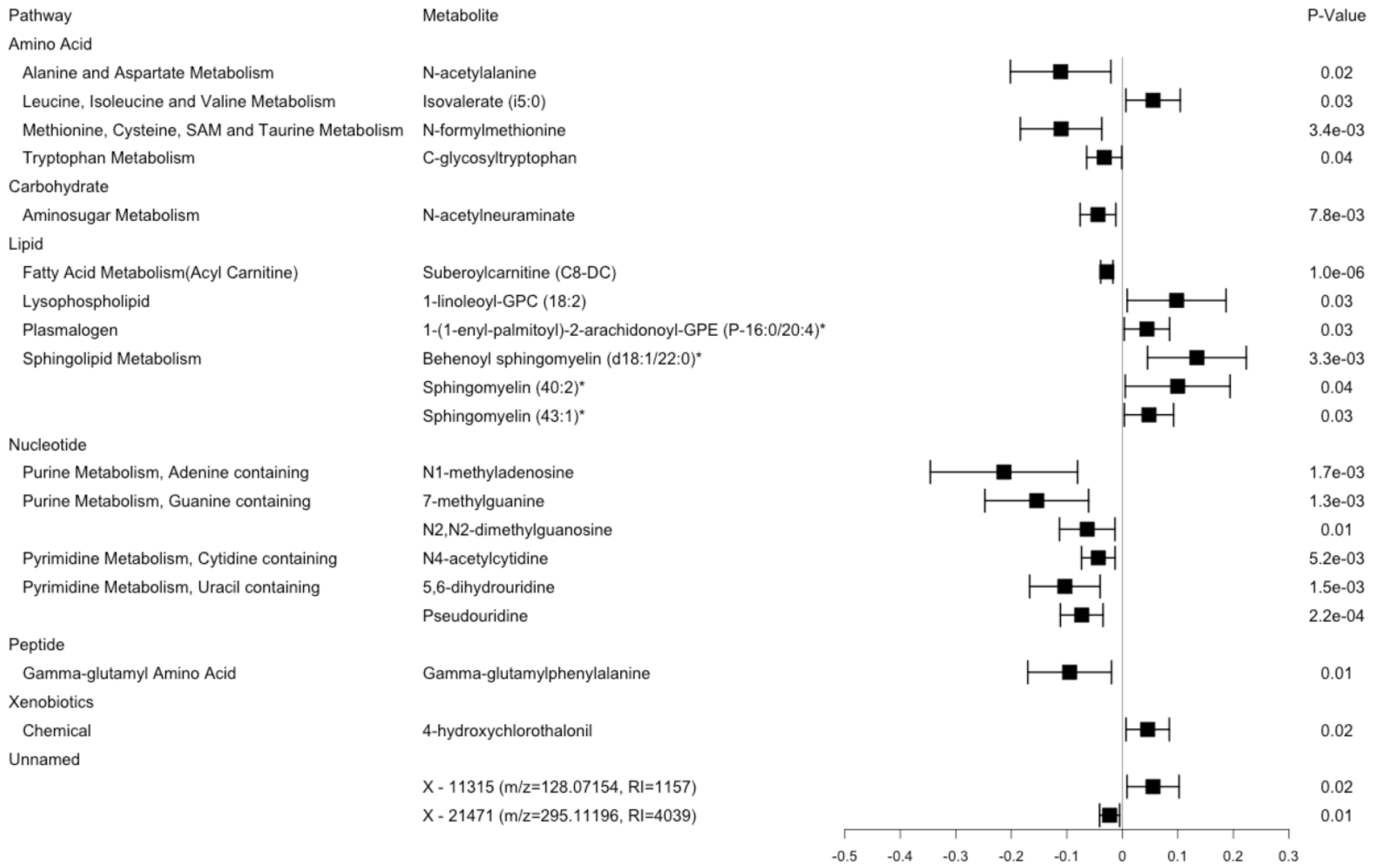

* Indicates compounds with Metabolomics Standards Initiative confidence level 2.

Supplementary Figure 6. Metabolites Significantly Associated with Gait Speed in Race Stratified Analysis, among Black Participants.

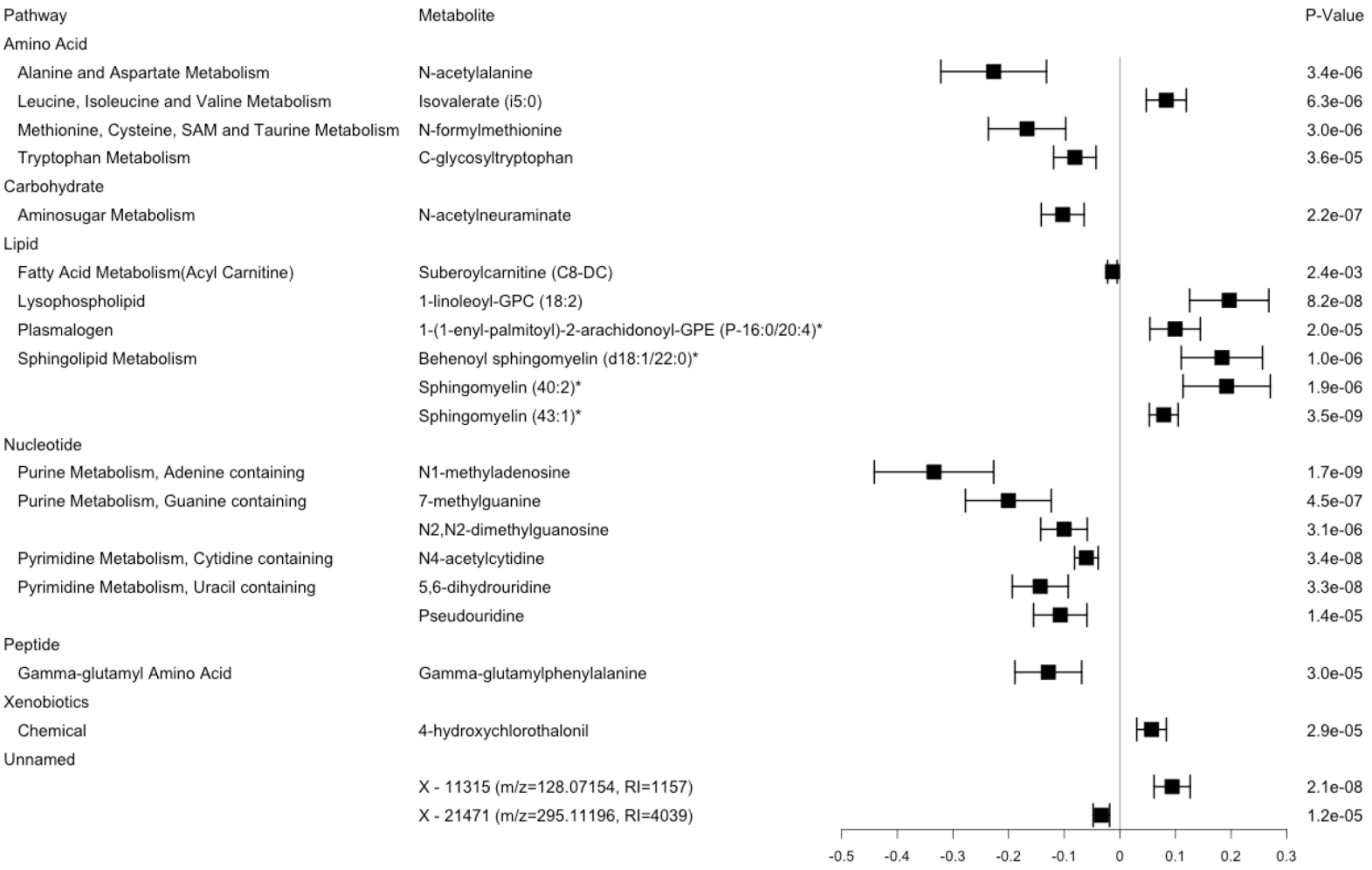

* Indicates compounds with Metabolomics Standards Initiative confidence level 2.

Supplementary Figure 7. Metabolites Significantly Associated with Gait Speed in Race Stratified Analysis, among White Participants.

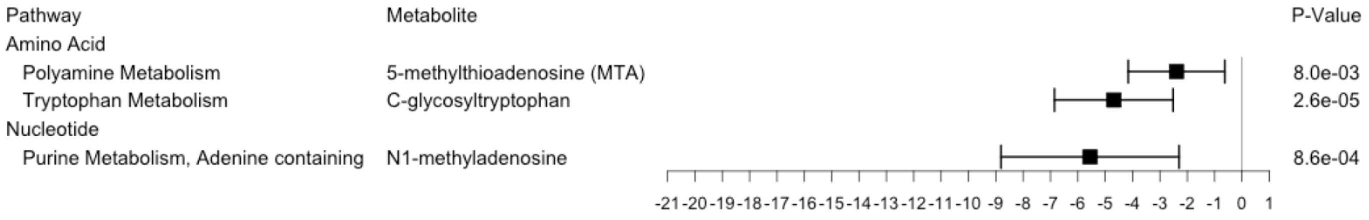

Supplementary Figure 8. Metabolites Significantly Associated with Grip Strength in Sex Stratified Analysis, among Female Participants.

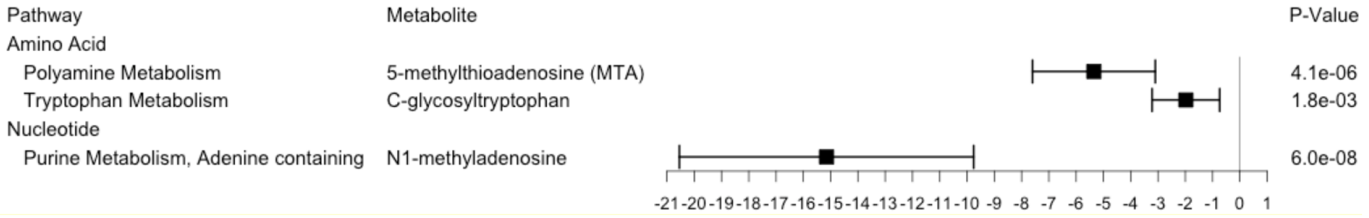

Supplementary Figure 9. Metabolites Significantly Associated with Grip Strength in Sex Stratified Analysis, among Male Participants.

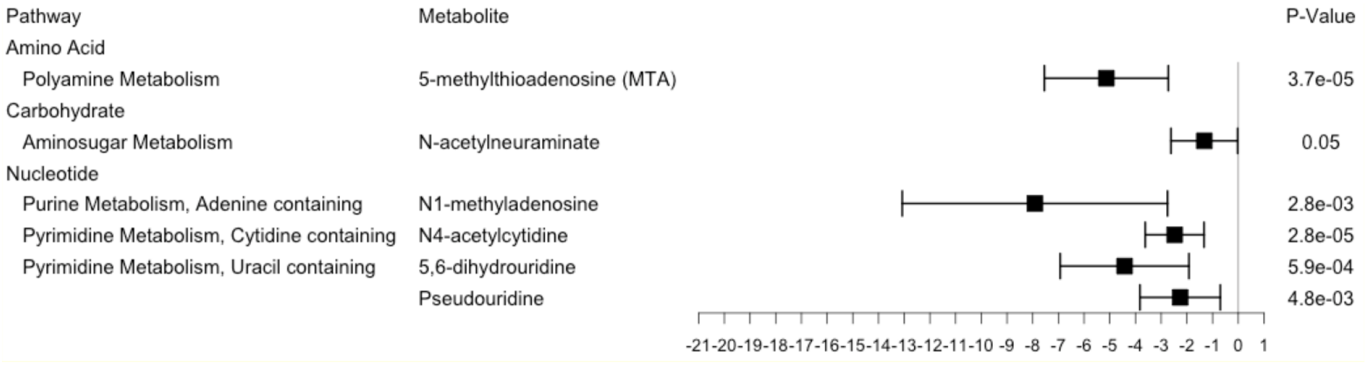

Supplementary Figure 10. Metabolites Significantly Associated with Grip Strength in Race Stratified Analysis, among Black Participants.

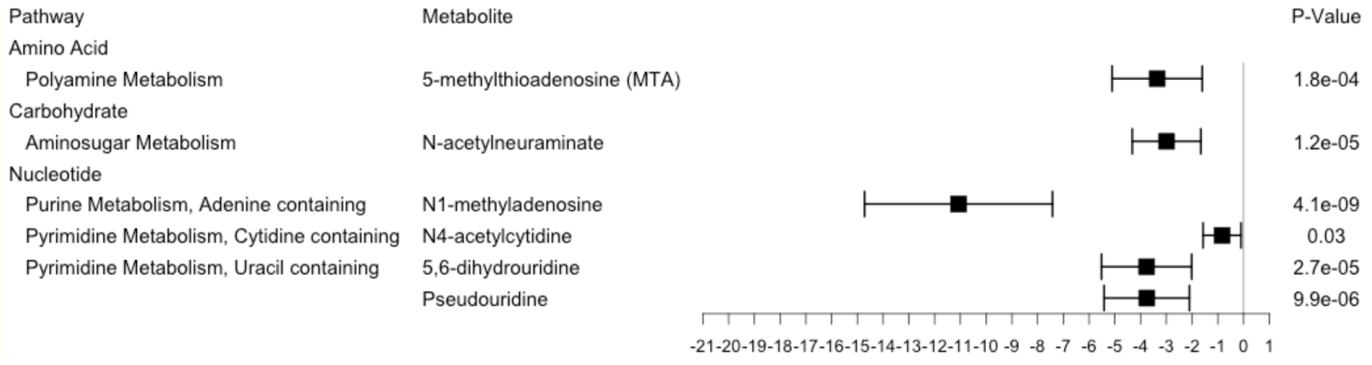

Supplementary Figure 11. Metabolites Significantly Associated with Grip Strength in Race Stratified Analysis, among White Participants.
