# Supplementary material for: Serum metabolites associate with physical performance among middle-aged adults: Evidence from the Bogalusa Heart Study
Source: Aging (Albany NY). 2020 Jun 1;12(12):11914–41. doi: 10.18632/aging.103362 (PMC7343486; doi:10.18632/aging.103362)
Supplement: Supplementary Tables [file aging-12-103362-s002..pdf]

SUPPLEMENTARY TABLES

Supplementary Table 1. Baseline characteristics of BHS Longitudinal Study Participants (N=288).

	Overall (N=288)	Female		Male	
		Black (n=87)	White (n=109)	Black (n=26)	White (n=66)
Age, years, mean (SD)	48.6 (5.2)	48.0 (5.4)	49.0 (4.9)	48.7 (5.6)	49.0 (5.1)
Post-high school education, n (%)	124 (43.1)	25 (28.7)	59 (54.1)	5 (19.2)	35 (53.0)
Smoking, n (%)					
Never	164 (56.9)	52 (59.8)	66 (60.6)	11 (42.3)	35 (53.0)
Former	88 (30.6)	23 (26.4)	33 (30.3)	8 (30.8)	24 (36.4)
Current	36 (12.5)	12 (13.8)	10 (9.2)	7 (26.9)	7 (10.6)
Drinking, n (%)					
Never	56 (19.4)	26 (29.9)	19 (17.4)	5 (19.2)	6 (9.1)
Former	87 (30.2)	26 (29.9)	32 (29.4)	9 (34.6)	20 (30.3)
Current	145 (50.4)	35 (40.2)	58 (53.2)	12 (46.2)	40 (60.6)
BMI, kg/m2, mean (SD)	31.0 (7.7)	34.6 (9.8)	29.3 (6.5)	29.8 (7.3)	29.6 (4.3)
SBP, mmHg, mean (SD)	121.7 (16.0)	123.4 (20.5)	116.3 (12.8)	129.0 (13.2)	125.6 (12.2)
Hypertension*, n (%)	172 (59.7)	62 (71.3)	49 (45.0)	21 (80.8)	40 (60.6)
Glucose, mg/dL, mean (SD)	104.9 (33.6)	105.8 (39.9)	103.6 (35.2)	105.7 (24.9)	105.4 (23.8)
Diabetes†, n (%)	49 (17.2)	16 (18.4)	18 (16.8)	8 (30.8)	7 (10.8)
eGFR, mL/min/1.73 m^2, mean (SD)	95.5 (17.1)	100.6 (19.6)	92.8 (13.7)	100.6 (17.2)	91.4 (16.6)
CKD (GFR‡<60 mL/min/1.73 m^2), n (%)	8 (2.8)	2 (2.3)	3 (2.8)	0 (0.0)	3 (4.6)
SPPB score	11.1 (1.4)	10.5 (1.8)	11.4 (1.0)	10.8 (2.0)	11.4 (0.9)
Six-minute walk distance (m)	419.4 (91.1)	369.3 (77.1)	430.5 (91.7)	422.0 (85.7)	464.6 (79.8)
Gait speed (m/s)	1.2 (0.3)	1.0 (0.2)	1.2 (0.3)	1.2 (0.2)	1.3 (0.2)
Grip strength (kg)	32.8 (11.6)	27.1 (6.6)	26.3 (6.1)	42.6 (9.2)	46.8 (9.5)

Supplementary Table 2. Gait Speed Metabolites from Main Findings in Sensitivity Analysis Additionally Adjusting for Fasting Glucose, Systolic Blood Pressure, and Low-Density Lipoprotein.

Pathway	Metabolite	Beta (SE)	P
Amino Acid			
Alanine and Aspartate Metabolism	N-acetylalanine	-0.17 (0.03)	4.7×10^{-7}
Alanine and Aspartate Metabolism	N-acetylaspartate (NAA)	-0.13 (0.02)	1.3×10-7
Glutathione Metabolism	2-aminobutyrate	0.09 (0.02)	2.3×10^{-7}
Leucine, Isoleucine and Valine Metabolism	Isovalerate (i5:0)	0.08 (0.02)	1.1×10^{-7}
Methionine, Cysteine, SAM and Taurine Metabolism	N-formylmethionine	-0.14 (0.03)	2.7×10^{-7}
Methionine, Cysteine, SAM and Taurine Metabolism	S-adenosylhomocysteine (SAH)	-0.02 (0.01)	9.7×10^{-5}
Phenylalanine Metabolism	1-carboxyethylphenylalanine	-0.04 (0.01)	2.0×10^{-6}
Polyamine Metabolism	N-acetyl-isoputreanine*	-0.05 (0.01)	5.00E-06
Tryptophan Metabolism	C-glycosyltryptophan	-0.05 (0.01)	4.90E-05
Carbohydrate			

Aminosugar Metabolism	N-acetylneuraminate	-0.06 (0.01)	7.6×10^{-7}
Cofactors and Vitamins			
Ascorbate and Aldarate Metabolism	Oxalate	0.06 (0.01)	6.9×10^{-9}
Lipid			
Fatty Acid Metabolism(Acyl Carnitine)	Suberoylcarnitine (C8-DC)	-0.02 (0.00)	1.0×10^{-7}
Lysophospholipid	1-linoleoyl-GPC (18:2)	0.17 (0.03)	5.1×10^{-8}
Plasmalogen	1-(1-enyl-palmitoyl)-2-arachidonoyl-GPE (P-16:0/20:4)*	0.07 (0.02)	4.4×10^{-6}
Sphingolipid Metabolism	Behenoyl sphingomyelin (d18:1/22:0)*	0.21 (0.04)	4.2×10^{-9}
Sphingolipid Metabolism	Sphingomyelin (40:2)*	0.18 (0.04)	2.2×10^{-6}
Sphingolipid Metabolism	Sphingomyelin (43:1)*	0.08 (0.01)	1.1×10^{-9}
Nucleotide			
Purine Metabolism, Adenine containing	N1-methyladenosine	-0.29 (0.04)	2.9×10^{-11}
Purine Metabolism, Adenine containing	N6-carbamoylthreonyladenosine	-0.05 (0.01)	1.0×10^{-4}
Purine Metabolism, Guanine containing	7-methylguanine	-0.18 (0.03)	2.6×10^{-8}
Purine Metabolism, Guanine containing	N2,N2-dimethylguanosine	-0.08 (0.02)	8.1×10^{-7}
Pyrimidine Metabolism, Cytidine containing	N4-acetylcytidine	-0.06 (0.01)	7.9×10^{-10}
Pyrimidine Metabolism, Uracil containing	5,6-dihydrouridine	-0.13 (0.02)	1.9×10^{-10}
Pyrimidine Metabolism, Uracil containing	Pseudouridine	-0.09 (0.02)	4.3×10^{-8}
Pyrimidine Metabolism, Uracil containing	Uridine	0.14 (0.03)	2.4×10^{-6}
Peptide			
Gamma-glutamyl Amino Acid	Gamma-glutamyl-2-aminobutyrate	0.06 (0.01)	1.9×10^{-7}
Gamma-glutamyl Amino Acid	Gamma-glutamylphenylalanine	-0.11 (0.02)	8.8×10^{-6}
Xenobiotics			
Bacterial/Fungal	Tartronate	0.07 (0.01)	1.7×10^{-9}
Chemical	4-hydroxychlorothalonil	0.06 (0.01)	2.9×10^{-7}
Food Component/Plant	Ergothioneine	0.06 (0.01)	1.5×10^{-7}
Food Component/Plant	Phytanate	0.04 (0.01)	1.5×10^{-7}
Unnamed			
	X - 11315 (m/z=128.07154, RI=1157)	0.09 (0.01)	6.8×10^{-10}
	X - 18914 (m/z=266.88894, RI=4503)	0.08 (0.02)	1.4×10^{-7}
	X - 21471 (m/z=295.11196, RI=4039)	-0.03 (0.01)	1.3×10^{-5}
	X - 24337 (m/z=239.07856, RI=1980)	-0.04 (0.01)	2.0×10^{-6}
	X - 24513 (m/z=149.05558, RI=1148)	-0.05 (0.01)	6.4×10^{-5}

SE=standard error. * Indicates compounds with Metabolomics Standards Initiative confidence level 2.

Supplementary Table 3. Grip Strength Metabolites from Main Findings in Sensitivity Analysis Additionally Adjusting for Fasting Glucose, Systolic Blood Pressure, and Low-Density Lipoprotein.

Pathway	Metabolite	Beta (SE)	P
Amino Acid			
Polyamine Metabolism	5-methylthioadenosine (MTA)	-3.79 (0.75)	5.7×10^{-7}
Tryptophan Metabolism	C-glycosyltryptophan	-2.08 (0.46)	6.5×10^{-6}
Carbohydrate			
Aminosugar Metabolism	N-acetylneuraminate	-2.09 (0.47)	9.5×10^{-6}
Nucleotide			
Purine Metabolism, Adenine containing	N1-methyladenosine	-10.05 (1.53)	8.3×10^{-11}
Pyrimidine Metabolism, Cytidine containing	N4-acetylcytidine	-1.2 (0.32)	2.0×10^{-4}
Pyrimidine Metabolism, Uracil containing	5,6-dihydrouridine	-4.08 (0.73)	3.1×10^{-8}
Pyrimidine Metabolism, Uracil containing	Pseudouridine	-3.05 (0.57)	1.1×10^{-7}

Supplementary Table 4. All associations with longitudinal change in gait speed between baseline and follow up.

Pathway	Metabolite	Beta (SE)	P-Value
Positive in Cross-Sectional Analysis			
Amino Acid			
Glutathione Metabolism	2-aminobutyrate	0.04 (0.03)	0.20
Leucine, Isoleucine and Valine Metabolism	Isovalerate (i5:0)	-0.01 (0.03)	0.75
Cofactors and Vitamins			
Ascorbate and Aldarate Metabolism	Oxalate	0.03 (0.02)	0.13
Lipid			
Lysophospholipid	1-linoleoyl-GPC (18:2)	0.06 (0.06)	0.36
Plasmalogen	1-(1-enyl-palmitoyl)-2-arachidonoyl-GPE (P-16:0/20:4)[a]	-0.02 (0.04)	0.55
Sphingolipid Metabolism	Behenoyl sphingomyelin (d18:1/22:0)[a]	0.15 (0.06)	0.02 *
	Sphingomyelin (40:2)[a]	0.26 (0.07)	2.6×10^{-4} **
	Sphingomyelin (43:1)[a]	0.05 (0.03)	0.07
Nucleotide			
Pyrimidine Metabolism, Uracil containing	Uridine	0.07 (0.06)	0.28
Xenobiotics			
Bacterial/Fungal	Tartronate	0.05 (0.03)	0.08
Chemical	4-hydroxychlorothalonil	-0.02 (0.02)	0.38
Food Component/Plant	Ergothioneine	-0.05 (0.02)	9.6×10^{-3}
	Phytanate	0.02 (0.02)	0.33
Unnamed			
	X - 11315 (m/z=128.07154, RI=1157)	0.01 (0.03)	0.70
	X - 18914 (m/z=266.88894, RI=4503)	0.05 (0.03)	0.16
Negative in Cross-Sectional Analysis			
Amino Acid			
Alanine and Aspartate Metabolism	N-acetylalanine	-0.12 (0.07)	0.08
	N-acetylaspartate (NAA)	-0.13 (0.06)	0.04 *
Methionine, Cysteine, SAM and Taurine	N-formylmethionine	-0.12 (0.05)	0.01 *

Metabolism			
	S-adenosylhomocysteine (SAH)	-0.03 (0.01)	0.02 *
Phenylalanine Metabolism	1-carboxyethylphenylalanine	-0.09 (0.02)	8.8×10^{-5} **
Polyamine Metabolism	N-acetyl-isoputreanine[a]	-0.03 (0.02)	0.15
Tryptophan Metabolism	C-glycosyltryptophan	-0.04 (0.02)	0.09
Carbohydrate			
Aminosugar Metabolism	N-acetylneuraminate	-0.05 (0.03)	0.04 *
Lipid			
Fatty Acid Metabolism(Acyl Carnitine)	Suberoylcarnitine (C8-DC)	-0.01 (0.01)	0.21
Nucleotide			
Purine Metabolism, Adenine containing	N1-methyladenosine	-0.08 (0.11)	0.44
Purine Metabolism, Adenine containing	N6-carbamoylthreonyladenosine	-0.04 (0.02)	0.06
Purine Metabolism, Guanine containing	7-methylguanine	-0.06 (0.08)	0.48
Purine Metabolism, Guanine containing	N2,N2-dimethylguanosine	-0.05 (0.03)	0.05 *
Pyrimidine Metabolism, Cytidine containing	N4-acetylcytidine	-0.02 (0.02)	0.21
Pyrimidine Metabolism, Uracil containing	5,6-dihydrouridine	-0.08 (0.04)	0.05
Pyrimidine Metabolism, Uracil containing	Pseudouridine	-0.06 (0.04)	0.09
Peptide			
Gamma-glutamyl Amino Acid	Gamma-glutamylphenylalanine	-0.12 (0.05)	0.03 *
Unnamed			
	X - 21471 (m/z=295.11196, RI=4039)	-0.03 (0.02)	0.10
	X - 24337 (m/z=239.07856, RI=1980)	-0.06 (0.02)	1.70E-03
	X - 24513 (m/z=149.05558, RI=1148)	-0.05 (0.02)	0.04

[a] Indicates compounds that have not been officially confirmed based on a standard.
* P<0.05.
** Significant at a Bonferroni corrected level for 33 tests ($P<1.5\times10^{-3}$).

Supplementary Table 5. All associations with longitudinal change in grip strength between baseline and follow up.

Pathway	Metabolite	Beta (SE)	P-Value
Negative in Cross-Sectional Analysis			
Amino Acid			
Polyamine Metabolism	5-methylthioadenosine (MTA)	0.97 (0.99)	0.33
Tryptophan Metabolism	C-glycosyltryptophan	-0.06 (0.55)	0.91
Carbohydrate			
Aminosugar Metabolism	N-acetylneuraminate	-0.13 (0.56)	0.82
Nucleotide			
Purine Metabolism, Adenine containing	N1-methyladenosine	-0.58 (2.18)	0.79
Pyrimidine Metabolism, Cytidine containing	N4-acetylcytidine	-0.07 (0.35)	0.84
Pyrimidine Metabolism, Uracil containing	5,6-dihydrouridine	-0.83 (0.87)	0.34
Pyrimidine Metabolism, Uracil containing	Pseudouridine	-0.90 (0.81)	0.27

Supplementary Table 6. Metabolites associated with both physical performance and kidney function.

Pathway	Metabolite	Phenotypes	Previous associations (PMIDs)		
			Aging	Inflammation	Mortality
Amino Acid					
Alanine and Aspartate Metabolism	N-acetylalanine	Gait Speed	26956554		
N-acetylaspartate (NAA)	Gait Speed	21422012			
Methionine, Cysteine, SAM and Taurine Metabolism	N-formylmethionine	Gait Speed	26975982	29390044	
S-adenosylhomocysteine (SAH)	Gait Speed	29291380			
Phenylalanine Metabolism	1-carboxyethylphenylalanine	Gait Speed			
Polyamine Metabolism	5-methylthioadenosine (MTA)	Grip Strength	8467864	29390044	
N-acetyl-isoputreanine*	Gait Speed				
Tryptophan Metabolism	C-glycosyltryptophan	Gait Speed and Grip Strength	23838602	26975982	29390044
Carbohydrate					
Aminosugar Metabolism	N-acetylneuraminate	Gait Speed and Grip Strength	26975982		
Lipid					
Fatty Acid Metabolism(Acyl Carnitine)	Suberoylcarnitine (C8-DC)	Gait Speed			
Nucleotide					
Purine Metabolism, Adenine containing	N1-methyladenosine	Gait Speed and Grip Strength	26975982		
N6-carbamoylthreonyladenosine	Gait Speed	26975982			
Purine Metabolism, Guanine containing	7-methylguanine	Gait Speed	2079962	29390044	
N2,N2-dimethylguanosine	Gait Speed	29390044			
Pyrimidine Metabolism, Cytidine containing	N4-acetylcytidine	Gait Speed and Grip Strength			
Pyrimidine Metabolism, Uracil containing	5,6-dihydrouridine	Gait Speed and Grip Strength			
Pseudouridine	Gait Speed and Grip Strength	533544	29390044		
Peptide					
Gamma-glutamyl Amino Acid	Gamma-glutamylphenylalanine	Gait Speed	26797767	24498130	
